# Synthesis of Antimicrobial Norlabdane Compounds with Rearranged Cycle B and Molecular Docking Studies

**DOI:** 10.3390/molecules29235714

**Published:** 2024-12-03

**Authors:** Alexandru Ciocarlan, Lidia Lungu, Sergiu Shova, Nicoleta Vornicu, Natalia Bolocan, Veaceslav Kulcitki, Aculina Aricu

**Affiliations:** 1 Institute of Chemistry, Moldova State University, 2028 Chisinau, Moldova; algciocarlan@yahoo.com (A.C.); lidilungu@yahoo.com (L.L.); natalia_secara@yahoo.com (N.B.); kulcitki@yahoo.com (V.K.); 2 “P. Poni” Institute of Macromolecular Chemistry, 700487 Iasi, Romania; shova@icmpp.ro; 3 Metropolitan Center of Research T.A.B.O.R. (Tehnologie Arta Biserica Ortodoxa Romana), 700066 Iasi, Romania; cmctaboriasi@yahoo.com

**Keywords:** (+)-sclareolide, methyl 7-oxo-13,14,15,16-tetranorlabd-8-en-12-oate, 7-oxo-12,13,14,15,16-pentanorlabd-8-ene, Baeyer-Villiger oxidation, antimicrobial activity, DFT

## Abstract

The synthesis of tetra- and pentanorlabdane compounds with rearranged cycle B based on commercially available (+)-sclareolide is reported. Desired compounds were prepared from intermediate ketones via Baeyer–Villiger oxidation. The structures of synthesized compounds were confirmed by spectral IR, 1D (^1^H, ^13^C, and DEPT), and 2D (H-COSY, H,C-HSQC, H,C-HMBC, H,N-HMBC, NOESY) NMR analyses, mass-spectrometry and single crystal X-rays diffraction. Two out of the four synthesized compounds showed high antifungal and antibacterial activities comparable to and exceeding standard antifungal (caspofungin) and antibacterial (kanamycin) agents. DFT calculations show that in gas and DCM, compound **4** is more stable than **3** with a difference in the Gibbs free energy of 23.3 kJ/mol and 20.7 kJ/mol, respectively. In water and methanol, compound **3** is slightly more stable, by 2.4 kJ/mol and 2.78 kJ/mol, respectively. Molecular docking to four targets DNA gyrase from *E. coli* (1KZN), Fabz from *P. aeruginosa* (1U1Z), dihydrofolate reductase from *C. albicans* (3QLS) and MurB from *E. coli* (2Q85) showed good agreement with the results of in vitro evaluation and confirmed the biological activity of compounds **3** and **4**, with binding affinities comparable and for some targets exceeding that of Caspofungin and Kanamycin.

## 1. Introduction

**Figure 1:**
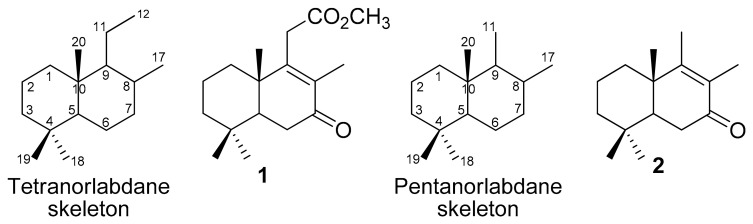
Tetra- and pentanorlabdane skeletons and starting ketones **1** and **2**.

It is well-known that natural pentanorlabdane lactones and some of the tetranorlabdane compounds possess a wide range of biological activity [1,2]. A large number of synthetic polyfunctional tetra- and pentanorlabdane biologically active compounds, including nitrogen-containing compounds, have been prepared, starting with (+)-sclareolide via related intermediate methyl 7-oxo-13,14,15,16-tetranorlabd-8-en-12-oate (**1**) and 7-oxo-12,13,14,15,16-pentanorlabd-8-ene (**2**) (drimenone) (Figure 1) [3,4,5,6,7,8,9,10,11].

These series can be completed by recently reported hybrid tetra- and pentanorlabdane heterocyclic compounds [12,13,14]. All these structures were obtained with the involvement of functional groups from the side chain or from cycle B of the initial molecules, without modifying their carbon skeleton. To date, only one synthesis of some lactams with the modified cycle B obtained by the Beckmann rearrangement of the oximes corresponding to compounds **1** and **2** has been reported [15].

The objective of this research was to capitalize on the synthetic opportunities presented by the initial compounds methyl 7-oxo-13,14,15,16-tetranorlabd-8-en-12-oate (**1**) and 7-oxo-12,13,14,15,16-pentanorlabd-8-ene (**2**), in particular for obtaining new compounds with the rearranged B ring as a result of Baeyer–Villiger oxidation. This was followed by their full spectral IR, 1D (^1^H, ^13^C, and DEPT), and 2D (H-COSY, H,C-HSQC, H,C-HMBC, H,N-HMBC, NOESY) NMR analyses, mass spectrometry and single-crystal X-rays diffraction, which proved the structures of synthesized compounds, antimicrobial assessment, and molecular docking.

## 2. Results and Discussion

### 2.1. Synthesis

The starting materials, methyl 7-oxo-13,14,15,16-tetranorlabd-8-en-12-oate (**1**) and drim-8-en-7-one (**2**) (drimenone), have traditionally been prepared synthetically due to their scarcity in natural sources [16]. They can be prepared by earlier known procedures in two and three steps, respectively, from commercially available (−)-sclareolide, in 60% and 57% yields [3,17], or by recently improved methods from the same starting material in comparable yields [18].

Baeyer–Villiger oxidation (BVO) has been widely and efficiently used for over a century for the conversion of ketones into lactones or esters [19], with some of these compounds being reported as biologically active. We tried to apply a modified procedure of BVO for terpenic ketones **1** and **2**, using 3-chloroperbenzoic acid (m-CPBA) combined with trifluoroacetic acid (TFA). Authors proved its efficiency for a series of cyclic, including *α*,*β*-unsaturated, and acyclic ketones, which were converted into corresponding esters and lactones in shorter times and high yields [20].

**Scheme 1:**
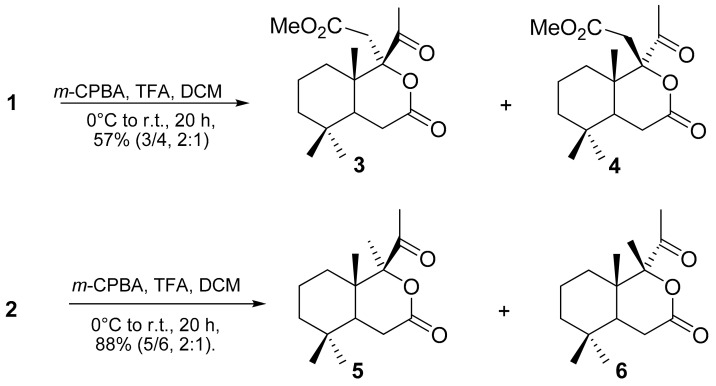
Baeyer-Villiger oxidation of norlabdane ketones **1** and **2**.

According to TLC data, the reaction mixture derived from ketoester **1** contained one major product, while that derived from drimenone **2** contained two chromatographically separable products. The NMR analysis showed that the unseparable mixture of compounds **3**/**4** and the individual compounds **5** and **6** are not seven-membered enol lactones, expected as the usual products of BVO. Surprisingly, the BVO reaction gave, in both cases, six-membered ring lactones **3**/**4** and **5**/**6** epimeric on C_9_, which were obtained in 57% and 88% overall yields, respectively (Scheme 1).

**Scheme 2:**
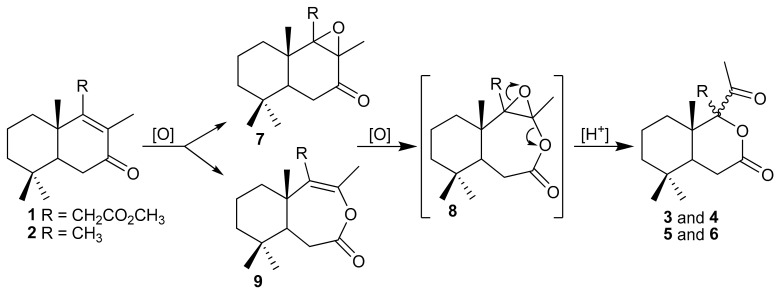
The proposed mechanism of the Baeyer–Villiger oxidation of norlabdane ketones **1** and **2**.

While the literature lacks reports of such rearrangements in norlabdane compounds during BVO, an explanation for the formation of compounds **3**–**6** exists. One of the ways of the ketones **1** and **2** conversion, probably, starts from epoxidation of the C_8-9_ double bond with epoxyketone **7** formation, followed by BVO, which leads to epoxylactone **8** (Scheme 2).

Another pathway of compound **8** formation starts with the BVO of ketones **1** and **2**, which gives enol lactone **9,** followed by the epoxidation of the double bond. Such types of transformations have been described for cholestane-type compounds, when both processes occur as a result of treating *α*,*β*-unsaturated ketones with peroxyacid [21].

However, in our case, as a result of both oxidative processes and due to acidic medium, a rearrangement of intermediate seven-membered ring epoxilactone **8** into a mixture of enantiomeric more stable six-membered lactones **3**/**4** and **5**/**6** occurs (Scheme 2).

### 2.2. Single-Crystal X-Ray Diffraction Study

**Figure 2:**
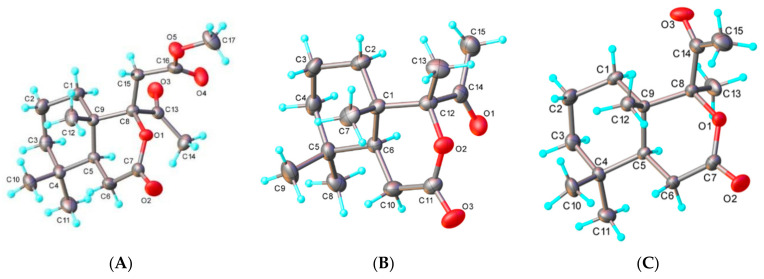
X-ray molecular structure of compounds **4** (**A**), **5** (**B**), and **6** (**C**) with atom labeling and thermal ellipsoids at 40% probability.

The structures of compounds **4**, **5**, and **6** were definitely confirmed by single-crystal X-ray diffraction. According to X-ray crystallography, they crystallize in the Sohnke *P*2_1_2_1_2_1_, *P*2_1_, and *P*2_1_2_1_2_1_ space groups, respectively, and exhibit a molecular crystal structure, where the asymmetric part consists of one neutral entity, as shown in Figure 2A–C, respectively. There are no co-crystallized solvate molecules in both crystals. The bond distances and angles are summarized in Table 1.

**Figure 3:**
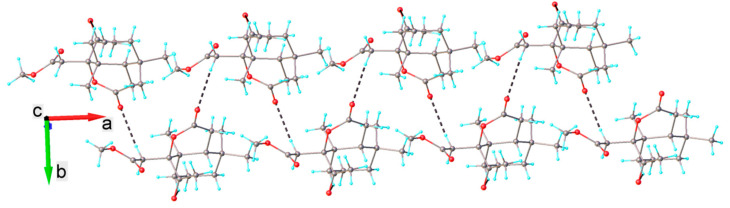
One-dimensional supramolecular chain in crystal structure **4**.

**Figure 4:**
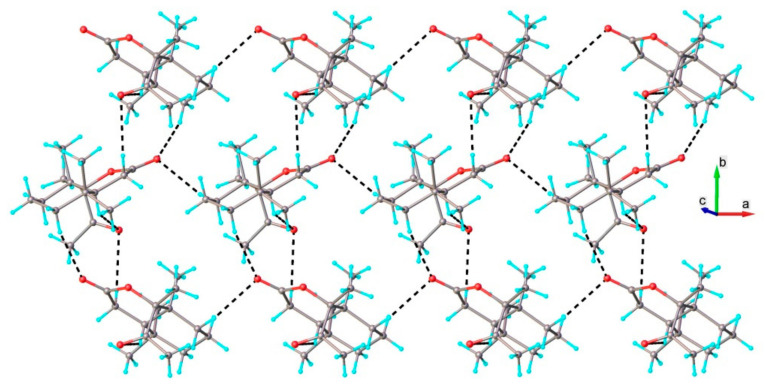
One-dimensional supramolecular chain in crystal structure **5**.

**Figure 5:**
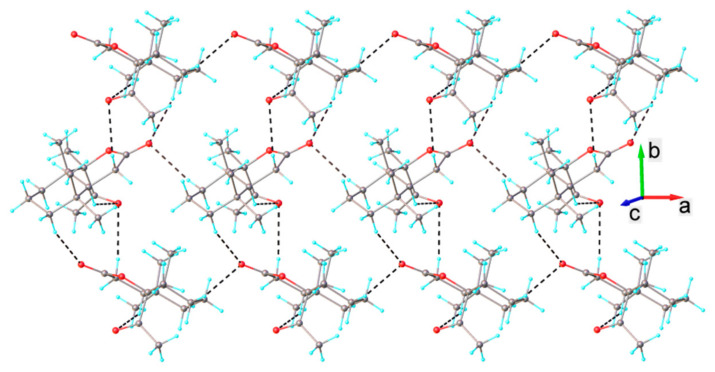
Two-dimensional supramolecular layer in crystal structure **6**.

Further analysis has revealed that the crystal structure of **4**, **5**, and **6** is driven by C-H---O hydrogen bonding. The aggregation of the molecules in crystal **4**, which occurs along hydrogen bonding, determines the formation of the discrete one-dimensional array having the form of a zigzag chain running along the *a* axis, as shown in Figure 3. The C-H---O hydrogen bonds in crystals **5** and **6**, more numerous but weaker, define the crystalline structure formed by the parallel packing of the two-dimensional network. A fragment of 2D supramolecular layer is shown in Figure 4 and Figure 5, respectively.

H-bond parameters: C15-H---O2 [C15-H 0.97 Å, H···O2(*x* − 0.5, 0.5 − *y*, −*z*) 2.565 Å, C15···O2 3.525(4) Å, ∠C15HO2 169.8°.

H-bond parameters: C6-H---O3 [C6-H 0.99 Å, H···O3(1 − *x*, 0.5 + *y*, 1 − *z*) 2.70 Å, C6···O3 3.553(5) Å, ∠C6HO3 144.1°; C15-H---O2 [C15-H 0.98 Å, H···O2(1 − *x*, *y* − 0.5, 1 − *z*) 2.83 Å, C15···O2 3.667(5) Å, ∠C15HO2 143.5°.

H-bond parameters: C2-H---O3 [C2-H 0.90 Å, H···O3(*x* − 1, *y*, *z*) 2.633 Å, C2···O3 3.572(4) Å, ∠C2HO3 158.4°; C4-H---O3 [C4-H 0.99 Å, H···O3(1 − *x*, *y* − 0.5, 1 − *z*) 2.654 Å, C4···O3 3.584(4) Å, ∠C4HO3 156.5°; C10-H---O1 [C10-H 0.99 Å, H···O1(1 − *x*, 0.5 + *y*, 1 − *z*) 2.704 Å, C10···O1 3.553(5) Å, ∠C10HO1 144.1°.

### 2.3. Antimicrobial Activity

The novel tetra- and pentanorlabdane compounds with rearranged cycle B **3**–**6** were preliminary screened for their in vitro antifungal and antibacterial activities against three pure cultures of fungi and two Gram-positive and Gram-negative bacteria strains (Table 1). Compounds **3** and **4** were inseparable from the racemic mixture and therefore were screened together. Compounds **5** and **6** did not show significant biological activity in the test.

According to the antimicrobial activity evaluation data, compounds **3** and **4** showed higher antifungal activity than the reference compound Caspofungin at MIC = 6.4·10^−2^ µg/mL, and comparable to the reference compound Kanamycin antibacterial activity at MIC = 2.0 µg/mL. The activity of compounds **3** and **4** is due to the co-existence of the lactone, >C=O, and ester groups.

### 2.4. DFT Study of the Structure of Compounds **3** and **4**

Given that compounds **3** and **4** showed good antifungal and antimicrobial activity, and that epimerizable product distribution in a reaction is controlled by their thermodynamic stability, the study continued with computational methods, in order to reveal details regarding the stability of these epimers in various media.

**Figure 6:**
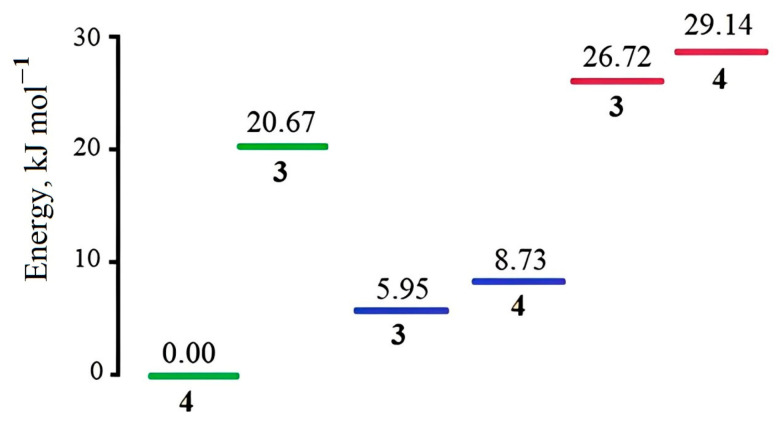
Relative energies of epimers **3** and **4** in DCM (green), methanol (blue), and water (red) calculated at the wB97X/def2-SVP level.

The structures of the two epimers **3** and **4** were optimized to global minima, in gas, water, DCM, and methanol, and the coordinates are presented in Supplementary Tables S1–S8. In gas, compound **4** is more stable than **3** with a difference in the Gibbs free energy of 23.3 kJ/mol. The relative energies of compounds **3** and **4** global minima in the studied solvents are shown in Figure 6.

As seen in Figure 6, compound **4** is more stable than compound **3** in DCM, with a difference in the Gibbs free energy of 20.7 kJ/mol. In water and methanol, compound **3** is slightly more stable, by 2.4 kJ/mol and 2.78 kJ/mol, respectively.

### 2.5. Molecular Docking of Compounds **3** and **4**

In order to explain the mechanisms of inhibition of microorganisms through the intermolecular ligand–receptor interaction that defines the antimicrobial activity, a comparative molecular docking study of epimers **3** and **4** was carried out on four protein models: DNA gyrase from *E. coli* (1KZN), Fabz from *P. aeruginosa* (1U1Z), dihydrofolate reductase from *C. albicans* (3QLS), and MurB from *E. coli* (2Q85).

The binding energies of the researched compounds (Table 2) are similar to those of the standards used, and, in many cases, they even exceed them, demonstrating the fact that they effectively bind to the target enzymes contributing to their inhibition. The results of computational calculations demonstrate the fact that most of the compounds have a higher binding affinity to 1U1Z, suggesting the idea that the interruption of fatty acid biosynthesis would be the most likely way of action of the investigated compounds on pathogens.

In the case of *E. coli* ADN girase (1KZN), Kanamycin has the highest binding affinity −6.8 kcal/mol, closely followed by compound **3**, (−6.6 kcal/mol), while compound **4** has a binding affinity very similar and a bit higher than Caspofungin, −6.2 kcal/mol and −6.1 kcal/mol, respectively. Both epimers bind into the same pocket as the standards, Caspofungin and Kanamycin (Supplementary Figure S1).

In binding to *P. aeruginosa* FabZ (1U1Z), compounds **3** and **4** have an activity of −7.7 kcal/mol, higher than Caspofungin (−7.3 kcal/mol), and close to that of Kanamycin (−8.0 kcal/mol). Both epimers bind into the same pocket as the standard Kanamycin (Supplementary Figure S2).

In the case of *C. albicans* dihidrofolat reductase (3QLS), Caspofungin and Kanamycin have very similar values of binding affinity, of −7.7 kcal/mol and −7.6 kcal/mol, respectively, while compounds **3** and **4** have lower affinities, of −6.7 kcal/mol and −6.4 kcal/mol, respectively. Both epimers bind into the same pocket as the standard Caspofungin (Supplementary Figure S3).

Lastly, in the case of binding to *E. coli* MurB (2Q85), Kanamycin has an affinity of −8.1 kcal/mol, followed by compound **3** with a binding affinity of −7.1 kcal/mol, and then Caspofungin and compound **4** with binding affinities of −6.8 kcal/mol and 6.4 kcal/mol, respectively. Both epimers bind into one pocket, different from that of Caspofungin and Kanamycin (Supplementary Figure S4).

The binding interactions of the synthesized tetranorlabdane lactones **3** and **4** with the amino acid residues of the target proteins (Table 3) are similar to a certain extent, consisting of two to five conventional hydrogen bonds, carbon–hydrogen bonds, and hydrophobic interactions.

## 3. Materials and Methods

The following reagents and solvents were used in the research: dichloromethane (DCM), trifluoroacetic acid (TFA), *meta*-chloroperbenzoic acid (*m*-CPBA), petroleum ether (PE), ethyl acetate (EtOAc), and deuterated chloroform (CDCl_3_). Reagents and solvents were purchased from Sigma-Aldrich (St. Louis, MO, USA).

Melting points (mp) were determined on a Boetius hot-stage apparatus (VEB Analytik, Göttingen, Germany). Optical rotations were measured on a Perkin-Elmer 241 polarimeter (Perkin-Elmer, Norwalk, CT, USA) with a 1 dm microcell, in CHCl_3_. Infrared spectra (IR) were obtained on Bio-Rad-Win-IR and Spectrum-100FT-IR (Perkin-Elmer) spectrometers with the ATR technique. ^1^H, and ^13^C NMR spectra were recorded in CDCl_3_ on Bruker Avance DRX 400 and Bruker Biospin Avance 500 spectrometers (Bruker BioSpin, Rheinstetten, Germany). Chemical shifts are given in parts per million values (ppm) in the *δ* scale and referred to CHCl_3_ (*δ*_H_ at 7.26 ppm) and to CDCl_3_ (*δ*_C_ at 77.00 ppm), respectively. Coupling constants (*J*) are reported in Hertz (Hz). Correlation spectroscopy (H,H-COSY), heteronuclear single quantum correlations (H,C-HSQC), and heteronuclear multiple-bond correlations (H,C-HMBC) experiments were recorded using standard pulse sequences, in the version with *z*-gradients, as delivered by Bruker Corporation. Carbon substitution degrees were established by distortionless enhancement by polarization transfer (DEPT) pulse sequence. GS-MS analyses were run on an Agilent-5975C model (Agilent, Santa Clara, CA, USA) provided a DB-17 fused silica capillary column, 30 × 0.25 mm i.d., with helium as the carrier gas, coupled with an Agilent-5975C spectrometer (EI, 70 eV). High-resolution mass spectra (MS) were run on a Kratos MS80 spectrometer (Kratos, Manchester, UK) (EI, 70 eV) with a home-built data system. The ratio *m*/*z* and relative intensity (%) are indicated for the significant peaks. For analytical thin-layer chromatography (TLC), Sorbfil (Krasnodar, Russia) and Merck (Rahway, NJ, USA) silica-gel plates 60 G in 0.25 mm layers were used. The chromatograms were sprayed with concentrated H_2_SO_4_ and heated at 80 °C for 5 min. Column chromatography was carried out on Across (Geel, Belgium) (60–200 mesh) and Merck silica gel 60 (70–230 mesh) using a mixture of petroleum ether (bp 40–60 °C) with EtOAc in gradient. All solvents were purified and dried by standard techniques before use. Organic extracts were dried over anhydrous Na_2_SO_4_, then filtered and evaporated under reduced pressure.

### 3.1. General Procedure of Baeyer–Villiger Oxidation of Ketones **1** and **2**

To a solution of *α*,*β*-unsaturated ketone (1 mmol) in DCM (4 mL), *m*-CPBA (2.2 mmol) was added. After cooling to 0 °C, freshly distilled TFA (0.076 mL) was added dropwise. In continuation, the reaction mixture was warmed to room temperature and stirred in the dark for 20 h, then diluted with DCM (20 mL) and washed consecutively with aqueous Na_2_SO_3_ (10%) (15 mL), saturated K_2_CO_3_ solutions, water (10 mL), and dried over anhydrous Na_2_SO_4_. After solvent removal under reduced pressure, the crude reaction products were purified by column chromatography on silica gel (eluent PE/EtOAc gradient 90/10→70/30) to yield:

Mixture of methyl 2-((1S,8aS)-1-acetyl-5,5,8a-trimethyl-3-oxooctahydro-1H-isochromen-1-yl)acetate (**3**) and methyl 2-((1R,8aS)-1-acetyl-5,5,8a-trimethyl-3-oxooctahydro-1H-isochromen-1-yl)acetate (**4**). White crystals (177 mg, 57%, in ~2:1 ratio). IR (ν, CCl_4_, cm^−1^): 2954, 1742, 1715, 1438, 1353, 1180, 1046, 816.

*Compound* **3**. ^1^H NMR (400 MHz, CDCl_3_, *δ*): 3.65 (s, 3H, CH_3_COO), 3.15 (d, 1H, *J* = 16.5, H-11), 2.82 (d, 1H, *J* = 16.5, H-11), 2.76 (dd, 1H, *J_1_* = 18.7, *J_2_* = 7.0, H-6), 2.58 (dd, 1H, *J_1_* = 19.3, *J_2_* = 12.6, H-6), 2.40 (s, 3H, H_17_), 1.09 (s, 3H, H-20), 0.93 (s, 3H, H-19), 0.83 (s, 3H, H-18). ^13^C NMR (100 MHz, CDCl_3_, *δ*): 209.5 (C=O), 170.3 (C_7_=O), 169.7 (C_12_), 93.9 (C_9_), 52.1 (CH_3_COO), 44.1 (C_5_), 40.8 (C_10_), 40.5 (C_3_), 38.7 (C_11_), 33.0 (C_4_), 32.3 (C_18_), 32.1 (C_1_), 30.6 (C_17_), 29.6 (C_6_), 21.3 (C_19_), 17.9 (C_2_), 15.7 (C_20_).

*Compound* **4**. ^1^H NMR (400 MHz, CDCl_3_, *δ*): 3.67 (s, 3H, CH_3_COO), 3.33 (d, 1H, *J* = 15.2, H-11), 2.82 (d, 1H, *J* = 15.8, H-11), 2.66 (dd, 1H, *J_1_* = 18.8, *J_2_* = 5.8, H-6), 2.45 (dd, 1H, *J_1_* = 18.4, *J_2_* = 13.1, H-6), 2.40 (s, 3H, H-17), 1.04 (s, 3H, H-20), 0.92 (s, 3H, H-19), 0.92 (s, 3H, H-18). ^13^C NMR (100 MHz, CDCl_3_, *δ*): 209.3 (C_8_), 169.4 (C_12_), 169.3 (C_7_=O), 93.8 (C_9_), 52.2 (CH_3_COO), 41.8 (C_5_), 40.9 (C_3_), 40.8 (C_10_), 40.8 (C_11_), 33.0 (C_4_), 32.8 (C_18_), 32.8 (C_1_), 30.6 (C_17_), 28.2 (C_6_), 21.4 (C_19_), 17.6 (C_2_), 15.6 (C_20_).

HR-EI-MS: Found 310.27802.C_17_H_26_O_5_ Calcd. 310.3843. *m/z* 310 (M^+^, 1), 279 (29), 249 (36), 218 (4), 193 (19), 165 (10), 145 (9), 133 (11), 121 (6), 109 24), 101 (8), 81 (15), 68 (4).

*(1S,8aS)-1-acetyl-1,5,5,8a-tetramethylhexahydro-1H-isochromen-3(4H)-one* (**5**). White crystals (83 mg, 33%), mp 133–134 °C (PE), ${\left[\alpha \right]}_{D}^{20}$ −102.6 (*c* 2.6, CHCl_3_). IR (*ν*, CCl_4_, cm^−1^): 2940, 1729, 1705, 1459, 1381, 1260, 1090, 825. ^1^H NMR (400 MHz, CDCl_3_, *δ*): 2.69 (dd, 1H, *J_1_* = 18.8, *J*_2_ = 6.7, H-6), 2.47 (dd, 1H, *J_1_* = 18.9, *J_2_* = 12.4, H-6), 2.28 (s, 3H, H-17), 1.40 (s, 3H, H-20), 1.08 (s, 3H, H-11), 0.93 (s, 3H, H-19), 0.83 (s, 3H, H-18). ^13^C NMR (100 MHz, CDCl_3_, *δ*): 209.7 (C=O), 171.3 (C_7_=O), 94.6 (C_9_), 44.1 (C_5_), 40.9 (C_3_), 40.0 (C_10_), 33.0 (C_1_), 32.9 (C_4_), 32.3 (C_18_), 29.6 (C_17_), 29.3 (C_6_), 21.2 (C_19_), 18.7 (C_20_), 18.1 (C_2_), 14.7 (C_11_). HR-EI-MS: Found 209.15444. C_15_H_24_O_3_ Calcd. 253.2513. *m/z* 209 (M^+^, −44, 44), 181 (6), 123 (100), 69 (20), 65 (2), 54 (1), 42 (3).

*(1R,8aS)-1-acetyl-1,5,5,8a-tetramethylhexahydro-1H-isochromen-3(4H)-one* (**6**). White crystals (137 mg, 55%), mp 90–91 °C (PE), ${\left[\alpha \right]}_{D}^{20}$ −73.6 (*c* 2.3, CHCl_3_). IR (*ν*, CCl_4_, cm^−1^): 2937, 1733, 1714, 1455, 1354, 1279, 1101, 827. ^1^H NMR (400 MHz, CDCl_3_, *δ*): 2.61 (dd, 1H, *J_1_* = 18.8, *J_2_* = 6.2, H-6), 2.41 (dd, 1H, *J_1_* = 18.8, *J_2_* = 13.2, H-6), 2.31 (s, 3H, H-17), 1.44 (s, 3H, H-11), 1.02 (s, 3H, H-20), 0.91 (s, 3H, H-19), 0.89 (s, 3H, H-18). ^13^C NMR (100 MHz, CDCl_3_, *δ*): 210.5 (C=O), 170.2 (C_7_=O), 95.0 (C_9_), 41.2 (C_5_), 41.0 (C_3_), 38.7 (C_10_), 33.1 (C_1_), 32.9 (C_4_), 32.7 (C_18_), 28.9 (C_17_), 28.4 (C_6_), 21.2 (C_20_), 21.2 (C_11_), 17.8 (C_2_), 15.7 (C_19_). HR-EI-MS: Found 209.15413. C_15_H_24_O_3_ Calcd. 253.2513. *m/z* 209 (M^+^, −44, 47), 181 (6), 110 (3), 107 (7), 95 (13), 91 (2), 83 (8), 79 (4), 67 (11), 59 (4), 55 (15), 51 (1).

### 3.2. Crystallographic Studies

Single-crystal X-ray diffraction data were collected on an Oxford-Diffraction XCALIBUR Eos CCD diffractometer (Agilent) with graphite-monochromated MoKα radiation. The unit cell determination and data integration were carried out using the CrysAlisPro package (version 1.171.43.137a) from Oxford Diffraction [22]. A multi-scan correction for absorption was applied. The structures were solved with the SHELXT using the intrinsic phasing method and refined by the full-matrix least-squares method on *F*^2^ with SHELXL (version 2018/3) [23,24]. Olex2 was used as an interface to the SHELX programs [25]. Non-hydrogen atoms were refined anisotropically. Hydrogen atoms were added in idealized positions and refined using a riding model. In the absence of a significant anomalous scattering, the absolute configuration of the structures could not be reliably determined, and therefore, Friedel pairs were merged and any references to the Flack parameter were removed. The molecular plots were obtained using the Olex2-1.5 program. Selected crystallographic data and structure refinement details for compounds **4**, **5,** and **6** are provided in Supplementary Table S9.

### 3.3. Antifungal and Antibacterial Activity Assay

The synthesized compounds were tested in vitro for antimicrobial activity on three species of fungi: *Aspergillus niger* (ATCC 53346), *Penicillium frequentans* (ATCC 10110), and *Alternaria alternate* (ATCC 8741), and both Gram-positive *Bacillus polymyxa* (ATCC 15970) and Gram-negative *Pseudomona aeruginosa* (ATCC 27813) bacteria strains, provided by the American Type Culture Collection (ATCC, Manassas, VA, USA). Compounds Caspofungin and Kanamycin, both from Liofilchem (Roseto degli Abruzzi, Italy), were used as standards for antifungal and antibacterial activity testing at (MIC = 0.38 µg/mL) and (MIC = 2.0 µg/mL), respectively. Microorganism suspensions were prepared using the method of successive agar dilutions according to the standard MIC [26], and their cultivation was carried out according to standard procedures [27].

### 3.4. DFT Calculations

The ORCA program (https://www.faccts.de/orca/, accessed on 29 October 2024) was used to optimize and properly characterize the structures [28]. All the calculations were performed using the wB97X functional with def2-SVP basis set, and the empirical dispersion correction of Grimme’s D3 in the DFT method [29]. Frequency analyses were performed at the same level of theory. All the calculations in solvents (water, dichloromethane, methanol) were performed by employing the Solvation Model Density calculation model (SMD).

### 3.5. Molecular Docking

Four protein models were used in the study: DNA gyrase from *Escherihia coli* (PDB code: 1KZN), FabZ from *P. aeruginosa* (PDB code: 1U1Z), dihydrofolate reductase from *Candida albicans* (PDB code: 3QLS), and MurB from *E. coli* (PDB code: 2Q85).

The structures were prepared according to the protocol, using the AutoDockTools 1.5.7 program: water molecules and native ligands were removed from the protein structures, and polar hydrogens and Gasteiger charges were added [30].

In the docking protocol, the ligands were treated as flexible molecules and the docking software was allowed to rotate all rotatable bonds of the ligands to obtain the ligand conformer with the optimal position in the active site of the enzyme. In each case, the grid was constructed and properly centered to cover the entire enzyme. Docking was performed using the routine procedure and default parameters of the molecular docking program Vina v.1.2.2 [31].

From the set of ligand conformations obtained by the docking procedure, the conformation with the lowest binding energy was selected for further analysis. The Discovery Studio Visualizer program (BIOVIA, San Diego, CA, USA, version 2021) was used to investigate the hydrophobic interactions and hydrogen bonds between the ligands and the investigated enzymes. The 3D structures of the Kanamycin and Caspofungin standards were retrieved from The Human Metabolome Database (https://hmdb.ca/).

## 4. Conclusions

A series of novel tetra- and pentanorlabdane six-membered lactones with rearranged cycle B were synthesized in good yields by the Baeyer–Villiger oxidation with m-CPBA and the mechanism of their formation was proposed.

Computational calculations revealed that epimer **4** is more stable in gas and DCM, by 23.3 kJ/mol and 20.7 kJ/mol, respectively, while epimer **3** is more stable in water and methanol, by 2.4 kJ/mol and 2.78 kJ/mol, respectively.

Molecular docking results are in good agreement with experimental findings, showing that compounds **3** and **4** have a biological activity comparable, and, in some cases, exceeding that of the standards Caspofungin and Kanamycin on our protein models: DNA gyrase from *E. coli* (1KZN), Fabz from *P. aeruginosa* (1U1Z), dihydrofolate reductase from *C. albicans* (3QLS), and MurB from *E. coli* (2Q85). Although the binding pattern of interactions of the synthesized tetranorlabdane lactones **3** and **4** with the amino acid residues of the target proteins are very similar, the values of calculated binding affinities show that epimer **3** has a higher biological activity than epimer **4**, which is in agreement with experimental data.

Due to their original structure and biological activity, the reported compounds are of real scientific interest.

## Figures and Tables

**Table 1:** Results of “in vitro” antimicrobial and antifungal activity of tested compounds.

Sample	MIC (µg/mL)				
	*Aspergillus niger*	*Penicillium* *frequentans*	*Alternaria* *alternate*	*Bacillus* *polymyxa*	*Pseudomonas* *aeruginosa*
**1/2**	>128	>128	>128	>128	>128
**3/4**	0.064 (±0.035)	0.064 (±0.035)	0.064 (±0.035)	2.0 (±0.060)	2.0 (±0.060)
Caspofungin *	0.38	0.38	0.38	-	-
Kanamycin *	-	-	-	2.0	2.0

* Standard compound.

**Table 2:** The binding energy of the studied compounds to the target enzymes.

Compound	Calculated Binding Energy (kcal/mol)			
	*E. coli* DNA Girase, 1KZN	*P. aeruginosa* FabZ, 1U1Z	*C. albicans* Dihidrofolate Reductase, 3QLS	*E. coli* MurB, 2Q85
**3**	−6.6	−7.7	−6.7	−7.1
**4**	−6.2	−7.7	−6.4	−6.4
Caspofungin	−6.1	−7.3	−7.7	−6.8
Kanamycin	−6.8	−8.0	−7.6	−8.1

**Table 3:** The binding interactions of the synthesized tetranorlabdane lactones **3** and **4** with the amino acid residues of the target proteins.

Protein	Compound 3		Compound 4	
	Type of Bonding	Amino Acid Residues	Type of Bonding	Amino Acid Residues
3QLS	Hydrogen bond	ASN5 ~2.3 Å	Hydrogen bond	ASN5 ~2.2 Å and ~2.8 Å
		ASN83 ~2.9 Å		ASN83 ~2.8 Å
		SER95 ~2.7 Å		SER94 ~2.1 Å
		SER98 ~2.3 Å		SER98 ~2.4 Å
	Carbon–hydrogen bond	PRO4 ~3.5 Å	Carbon-hydrogen bond	GLU82 ~3.6 Å
		SER95 ~3.7 Å		
	Hydrophobic (alkyl)	ARG108 ~4.5 Å		
2Q85	Hydrogen bond	ARG159 ~2.6 Å and ~1.8 Å	Hydrogen bond	ARG159 ~2.8 ÅSER229 ~2.2 Å
		SER229 ~2.4 Å		
		ASN233 ~2.0 Å		
	Hydrophobic (alkyl)	ALA124 ~4.2 Å	Hydrophobic (alkyl)	LEU218 ~4.4 Å
			Carbon-hydrogen bond	PHE231 ~3.5 Å
				GLU325 ~3.5 Å
1U1Z	Hydrogen bond	TYR15 ~2.4 Å and ~2.5 Å	Hydrogen bond	TYR15 ~2.0 Å and ~1.9 Å
	Carbon–hydrogen bond	TYR15 ~3.7 Å	Carbon-hydrogen bond	PRO44 ~3.6 Å
	Hydrophobic (Pi-alkyl)	TYR15 ~5.3 Å	Hydrophobic (Pi-alkyl)	TYR15 ~5.6 Å
1KZN	Hydrogen bond	ARG76 ~3.0 Å	Hydrogen bond	ASN46 ~2.0 ÅTHR165 ~2.4 Å
		ARG136 ~3.0 Å		
		THR165 ~2.4 Å		
	Hydrophobic (alkyl)	ILE78 ~5.1 Å	Carbon-hydrogen bond	ILE78 ~3.4 Å
		ILE90 ~5.0 Å		PRO79 ~3.3 Å

## Data Availability

Data presented in this study are available on request from the corresponding author.

## Supplementary Materials

The following supporting information can be downloaded at: https://www.mdpi.com/article/10.3390/molecules29235714/s1, Table S1: Coordinates of epimer **3** in gas. Table S2: Coordinates of epimer **4** in gas. Table S3: Coordinates of epimer **3** in water. Table S4: Coordinates of epimer **4** in water. Table S5: Coordinates of epimer **3** in dichloromethane. Table S6: Coordinates of epimer **4** in dichloromethane. Table S7: Coordinates of epimer **3** in methanol. Table S8: Coordinates of epimer **4** in methanol. Figure S1: Positions of the best score conformations in the binding pocket of 1KZN (Caspofungin—yellow, Kanamycin—purple, compound **3**—blue, compound **4**—gray). Figure S2: Positions of the best score conformations in the binding pocket of 1U1Z (Caspofungin—yellow, Kanamycin—purple, compound **3**—blue, compound **4**—gray). Figure S3: Positions of the best score conformations in the binding pocket of 3QLS (Caspofungin—yellow, Kanamycin—purple, compound **3**—blue, compound **4**—gray). Figure S4: Positions of the best score conformations in the binding pocket of 2Q85 (Caspofungin—yellow, Kanamycin—purple, compound **3**—blue, compound **4**—gray). Table S9: Crystal data and details of structure refinement for **4**, **5** and **6**. Figure S5: The IR spectrum of compounds **3** and **4**. Figure S6: The 1H NMR spectrum of compounds **3** and **4**. Figure S7: The 13C NMR spectrum of compounds **3** and **4**. Figure S8: The IR spectrum of compound **5**. Figure S9: The 1H NMR spectrum of compound **5**. Figure S10: The 13C NMR spectrum of compound **5**. Figure S11: The IR spectrum of compound **6**. Figure S12: The 1H NMR spectrum of compound **6**. Figure S13: The 13C NMR spectrum of compound **6**.
