# Supplementary material for: Synthesis of Antimicrobial Norlabdane Compounds with Rearranged Cycle B and Molecular Docking Studies
Source: Molecules. 2024 Dec 3;29(23):5714. doi: 10.3390/molecules29235714 (PMC11643738; doi:10.3390/molecules29235714)
Supplement: Supplementary file 1 [file molecules-29-05714-s001.zip › molecules-3314882-supplementary.pdf]

Synthesis of Antimicrobial Norlabdane Compounds with Rearranged Cycle B and Molecular Docking Studies

Alexandru Ciocarlan [1], Lidia Lungu [1], Sergiu Shova [2], Nicoleta Vornicu [3], Natalia Bolocan [1], Veaceslav Kulcitki [1] and Aculina Aricu [1,*]

[1] Institute of Chemistry, Moldova State University, 2028 Chisinau, Moldova; algciocarlan@yahoo.com (A.C.); lidilungu@yahoo.com (L.L.); natalia_secara@yahoo.com (N.B.); kulcitki@yahoo.com (V.K.)
[2] "P. Poni" Institute of Macromolecular Chemistry, 700487 Iasi, Romania; shova@icmpp.ro
[3] Metropolitan Center of Research T.A.B.O.R. (Tehnologie Arta Biserica Ortodoxa Romana),
700066 Iasi, Romania; cmctaboriasi@yahoo.com
* Correspondence: aculina.aricu@gmail.com; Tel.: +373-691-86-602

Supporting Information

Table S1: Coordinates of epimer **3** in gas.

C	-5.43821419247360	-0.06639306002008	1.99574537951651
C	-5.47484487144424	-1.58394456716042	1.94842604888440
C	-5.11467626811834	-2.13427562713950	0.55872720579242
C	-3.69087675701584	-1.61227384509741	0.17070131355947
C	-3.29798049202322	-0.11408136570167	0.45388056146411
C	-4.02138859796922	0.42653668388699	1.71766688367487
C	-3.29521321710836	-1.96880174633428	-1.26100517773554
C	-3.99216569274552	-1.10855138958109	-2.28051908609550
O	-4.22955237187904	0.16346871230237	-1.89826841801007
C	-3.50256325740307	0.80045853416861	-0.85383913462926
C	-1.79742951551499	-0.14055542946669	0.83386859937820
C	-4.19792579871295	2.16426489618499	-0.64740042113143
O	-4.34660387469811	-1.47416969896520	-3.36410599153232
C	-2.13842262432527	1.28044425198611	-1.47020186399118
O	-1.47701651793331	2.09515394841130	-0.87465518695821
C	-1.74342151930509	0.79903780236214	-2.84362181235175
C	-5.04513589004276	-3.66672971005229	0.67046546401515
C	-6.22211163026071	-1.78340434999327	-0.44709465708664
C	-5.65501765710169	2.20338920916833	-0.23043454806332
O	-6.49843904658815	1.48316482016767	-0.68308744901290
O	-6.02564793939076	3.14207496715326	0.65965476946576
C	-5.15239735223037	4.12886674401395	1.16987832076738
H	-5.74067536875969	0.29232296605443	2.99250288688151
H	-6.17348576392421	0.34061512956114	1.29086413385963
H	-6.47204955134383	-1.95626717400114	2.23477252224255
H	-4.76553922432988	-1.99103397265850	2.69355593029615
H	-3.02276006573315	-2.18933846284915	0.83036099593935
H	-3.41091038174534	0.10864858882520	2.57887412362809
H	-3.96799263165682	1.52472753632168	1.74053542950944
H	-2.20872924894248	-1.83818046909404	-1.39623061543435

H	-3.50676226008196	-3.01298229202032	-1.52062470705216
H	-1.42851395184298	0.85560667044078	1.10767663995061
H	-1.65962294678604	-0.81081485406609	1.69348060219109
H	-1.15247655485342	-0.51146662507486	0.02231219252201
H	-4.18314715100545	2.67029666803356	-1.62817874124792
H	-3.54720845197090	2.75461027511508	0.00394900774112
H	-1.57182491844925	-0.28694294668924	-2.86165266268136
H	-2.54563765981850	0.99851360224795	-3.56825523337275
H	-0.82205492926239	1.31535307601958	-3.13611764834672
H	-4.82844749767922	-4.14882393764294	-0.29488139917945
H	-6.01197604444550	-4.06318641665455	1.01736423552128
H	-4.27626415814390	-3.98719415991892	1.39224227480575
H	-6.06886711503882	-2.28895841121474	-1.41221847368178
H	-6.31312554647918	-0.70851602271641	-0.64684876681649
H	-7.19133731109451	-2.13389751399887	-0.05856593938641
H	-5.76865203606194	4.78145048449283	1.79950959576084
H	-4.35524769782882	3.69308982304421	1.79340799376703
H	-4.70114644843644	4.73508865814953	0.36948482266279

Table S2: Coordinates of epimer **4** in gas.

C	-5.31668901258687	-0.65030462022353	2.25900595707542
C	-4.82209227730030	-2.08533155809104	2.16157620342707
C	-4.12437359725303	-2.38704071336301	0.82333318309571
C	-2.99115536130214	-1.32705838787079	0.57003121020310
C	-3.23695626428650	0.18245583438057	0.93195972868056
C	-4.12381567725083	0.29126511803516	2.19731330698683
C	-2.40584435143479	-1.40698856334150	-0.84203597598253
C	-3.35703315471032	-0.85646310942774	-1.87449610860578
O	-4.18626413284057	0.10991390831685	-1.42387574482570
C	-3.80063762727317	0.97365394558472	-0.36010534253359
C	-1.87300132400393	0.76377021875594	1.36733137633981
C	-2.77982428586485	1.97336403143190	-0.93980423798397
O	-3.45244698629612	-1.24967209231973	-3.00053680363613
C	-5.05038412110019	1.81505760389848	0.00528166503488
O	-4.87860237288090	2.79909936893754	0.68514752055558
C	-6.40042110390421	1.40313791882749	-0.50698639903454
C	-3.45447683536561	-3.76663565796959	0.94184955454482
C	-5.19370774787567	-2.49251933823985	-0.27642011622095
C	-3.33001412263543	2.80032030725538	-2.09445647574798
O	-4.48047522903831	2.83204914359189	-2.42337238560433
O	-2.44228123877075	3.55320308056043	-2.76520131290851
C	-1.07111454631633	3.60405886477915	-2.43510645856050
H	-5.84836817537535	-0.49513856268613	3.21085764254191
H	-6.05081790425172	-0.43551600576317	1.46756926001672
H	-5.64721369875244	-2.80284266124853	2.30222735928753
H	-4.11179462599128	-2.26803429811210	2.98866173413088
H	-2.19319461423735	-1.63816695955363	1.26432484492947
H	-3.47450495768435	0.02533879376819	3.04839554001997
H	-4.41228762512756	1.33620503047793	2.36538024444812
H	-1.46569970468103	-0.83413777348249	-0.90119834526660
H	-2.15203009368499	-2.42889760730348	-1.15090268525372
H	-1.97329127953176	1.80907942188239	1.69387014042454
H	-1.09900087310293	0.71943920990699	0.58886998915661

H	-1.49846370868192	0.19155634801913	2.22744012996485
H	-2.42324686621234	2.66041926390135	-0.16420334020011
H	-1.91745491901087	1.42328000571457	-1.34592094121348
H	-6.44524622175842	1.71550082875341	-1.55969686789087
H	-6.55155832853264	0.31773220471392	-0.48649331342451
H	-7.17404572010279	1.92403447963494	0.06968563549621
H	-2.96785421162669	-4.07621496272904	0.00427654760857
H	-4.20470203044636	-4.53519886363465	1.18410264556301
H	-2.69505407162320	-3.77858037135944	1.73991549236169
H	-5.93196275477794	-3.25587915091578	0.01322589369236
H	-4.77527298680003	-2.80719712577044	-1.24313040135408
H	-5.73543720791971	-1.55441527924694	-0.44395311874786
H	-0.62397362508069	4.35097034346010	-3.10150021586663
H	-0.56993561420283	2.63786375350013	-2.60677736238583
H	-0.90610921751184	3.91910478656403	-1.39281170033805

Table S3: Coordinates of epimer **3** in water.

C	-5.33472360265746	0.02456962024975	1.98267035200778
C	-5.46290299024968	-1.48943129085882	1.96068133431642
C	-5.12349793191774	-2.08812308449714	0.58559847393927
C	-3.69134977507980	-1.61752279178746	0.16255090064000
C	-3.23565953770851	-0.13558199046999	0.42965373658362
C	-3.88629026943582	0.41523003352779	1.72021476919995
C	-3.33328739926502	-2.00221854031396	-1.27179648855283
C	-3.99762969937495	-1.11603051721127	-2.27555877618936
O	-4.17647789414233	0.16393677392192	-1.92367783684676
C	-3.46787006766489	0.80522309318157	-0.85280594359259
C	-1.72232216108455	-0.21681269847852	0.74266911947524
C	-4.19732059726776	2.15092183441888	-0.62969891261623
O	-4.40401263446889	-1.46841797651854	-3.35982940476165
C	-2.11557402968419	1.31266237804221	-1.45839064629319
O	-1.47614135700774	2.14066488933457	-0.84582975064593
C	-1.69217263218461	0.83391918936069	-2.81471712067593
C	-5.09262080303600	-3.61714077513843	0.73605713630625
C	-6.23318741697541	-1.74221666411557	-0.41639236809031
C	-5.68492740327440	2.18447535919925	-0.37272213833989
O	-6.49043357643488	1.52010774494552	-0.98659740768796
O	-6.16163911032029	3.06566836509059	0.50924329296210
C	-5.34821063340141	4.00073582901350	1.21586958609885
H	-5.63583959084988	0.42143750828573	2.96496171488847
H	-6.03122844624609	0.46030838048526	1.25351241334302
H	-6.48114169935657	-1.80237611165934	2.24515745505250
H	-4.77902864785379	-1.91637156621814	2.71653839438274
H	-3.03026029385448	-2.21033329071428	0.81221717181720
H	-3.29980019724658	0.00360535729837	2.55767740686238
H	-3.73726941941839	1.50181690735330	1.78089101543026
H	-2.24571686083225	-1.91142897249302	-1.42646595303845
H	-3.59224535948583	-3.03816782088948	-1.52211697387854
H	-1.32537638972053	0.75271843824476	1.06802165149064
H	-1.56807713615097	-0.93596061103323	1.55856336991032
H	-1.11929940286675	-0.55531011767537	-0.11393606465175
H	-4.10079315664088	2.72708841435700	-1.56667141626788
H	-3.62949574246278	2.70770633802247	0.11914125561496

H	-1.55079578096764	-0.25660970927895	-2.83126162941110
H	-2.46644103877790	1.07476437015030	-3.55748788211538
H	-0.74964913968191	1.32511482134995	-3.08292663358872
H	-4.89302118072594	-4.12691533524055	-0.21916305210202
H	-6.06534378789574	-3.98242991226751	1.10226564456829
H	-4.32293503689402	-3.93477766048583	1.45755164500110
H	-6.09084431561178	-2.25998088925523	-1.37724849700259
H	-6.31322122709689	-0.66810740998143	-0.62115529328442
H	-7.20407530002772	-2.07851370916350	-0.01884581787986
H	-6.04267046268920	4.62771500487751	1.78636497212058
H	-4.67096447188438	3.49201257021003	1.91608265602466
H	-4.77771439212394	4.63477622482465	0.52324053947676

Table S4: Coordinates of epimer **4** in water.

C	-5.42187694701428	-0.03884927426989	1.98891982925257
C	-5.47247502049706	-1.55639244272525	1.95197179378805
C	-5.11792035954072	-2.12186147342172	0.56720088952694
C	-3.69964997328956	-1.60428812888095	0.15721043887271
C	-3.29928645952136	-0.10809160569285	0.43465849100905
C	-4.00169588777110	0.43660704452469	1.70488038750249
C	-3.32005982644291	-1.97658392611150	-1.27447371969526
C	-3.99887339553151	-1.10295251983017	-2.27956275717427
O	-4.18904182528237	0.17633920464568	-1.93269179704939
C	-3.48102814533106	0.82879130254704	-0.85488265324499
C	-1.79359077523003	-0.14097368786924	0.79655751419987
C	-4.22351069525578	2.17433983376897	-0.69314131640473
O	-4.40129881278856	-1.46450589312236	-3.36248460206179
C	-2.12712533057343	1.31545770228118	-1.48135091473714
O	-1.50436346636951	2.18928789306970	-0.91731217474176
C	-1.67992737876434	0.77274410488160	-2.80648810306267
C	-5.04877141466813	-3.65173127359047	0.69361556862603
C	-6.23036781193564	-1.78411339535254	-0.43472339310320
C	-5.66382937387274	2.20032723100273	-0.24842841983021
O	-6.52625520900451	1.51701972128149	-0.75646816147309
O	-6.03949895317309	3.07326113521527	0.68671918864067
C	-5.17065277118250	4.06191183567785	1.24032798537061
H	-5.71822125108310	0.32897451781170	2.98400730135603
H	-6.15661031160309	0.36854396025659	1.28277355127902
H	-6.47151931640823	-1.92127706279696	2.24255123911264
H	-4.76120027491410	-1.95972189021633	2.69557307060612
H	-3.02420909960910	-2.17664760651548	0.81069826672726
H	-3.39209273051911	0.09525970491855	2.55681915656612
H	-3.92331381658538	1.53213319283332	1.73698369686191
H	-2.23355699138276	-1.86228491074827	-1.41943482666743
H	-3.55374343728180	-3.01759875800156	-1.52774852647064
H	-1.43103859655806	0.85000388180510	1.09939327215812
H	-1.64381414688623	-0.82866290082814	1.63985899453800
H	-1.15862163689468	-0.49137796484529	-0.03195996870918
H	-4.24897263227498	2.63718940322716	-1.69419309887389
H	-3.58684524866456	2.81938882393314	-0.08126996144317
H	-1.52255925776248	-0.31431861188258	-2.76708099106972
H	-2.44258104077474	0.96909878030749	-3.57352679222968
H	-0.73905836689782	1.26253388885196	-3.08262009047350

H	-4.83713253926545	-4.14341176536275	-0.26832371976992
H	-6.01247389814375	-4.04516669521066	1.05439625993004
H	-4.27209868652110	-3.96122643709514	1.41121572996178
H	-6.08141068804961	-2.29936493187054	-1.39585560206389
H	-6.31634162752745	-0.70981986035653	-0.63705059323952
H	-7.19975351041123	-2.12778263068477	-0.03960369637445
H	-5.79029318459970	4.64994074986786	1.92633513854412
H	-4.34609720927087	3.60459262784158	1.80487425135506
H	-4.77684066707008	4.72165910673130	0.45523386417829

Table S5: Coordinates of epimer **3** in dichloromethane.

C	-5.40611154385364	-0.04371193203289	1.99463243430263
C	-5.47030055388274	-1.56086870695993	1.95429734428745
C	-5.11612536098505	-2.12468174881254	0.56821191372514
C	-3.69051063935394	-1.61754050699390	0.16608949302790
C	-3.27898797309391	-0.12359045344832	0.44713700153185
C	-3.97983507294225	0.42047759711594	1.71997340591433
C	-3.30900359810231	-1.98148188484001	-1.26819736237941
C	-4.00075923247227	-1.11236040204457	-2.27594681475025
O	-4.21032472757239	0.16208316839423	-1.90829706867834
C	-3.49026297309062	0.80270604901640	-0.85176961316523
C	-1.77452343227602	-0.17008078095129	0.80711565200343
C	-4.20003496189596	2.15783348788407	-0.63884791556914
O	-4.39205925768163	-1.47565403947949	-3.35494499779248
C	-2.12960868509723	1.29594585940810	-1.46246689598376
O	-1.47765717224183	2.11566845771922	-0.85946654021626
C	-1.72190017508313	0.81798668086070	-2.82882045033779
C	-5.06089872811986	-3.65619585180384	0.68870149743555
C	-6.22434912691843	-1.77197238339092	-0.43522011012225
C	-5.66714433705403	2.19669444093957	-0.27210758623532
O	-6.50649200291002	1.49481215333140	-0.77660692780683
O	-6.06879236760183	3.11688949378751	0.61196188427204
C	-5.20788319622307	4.10231016213280	1.16913543834012
H	-5.70757391298882	0.32568727350063	2.98812625850330
H	-6.12995829438426	0.37226904295719	1.28189336747991
H	-6.47390379838136	-1.91717068716755	2.24077053995346
H	-4.76593924577497	-1.97236837668565	2.70063690461980
H	-3.02260764315927	-2.20009443639472	0.81922322304917
H	-3.37549325519898	0.07343280339148	2.57387031656999
H	-3.89948485528569	1.51621037009616	1.75636465455073
H	-2.22323884589307	-1.85565951815124	-1.41121186296667
H	-3.53224922227878	-3.02457725559468	-1.52333888598485
H	-1.39806551841256	0.81689289538914	1.10413923363173
H	-1.63310199085419	-0.85889740666923	1.65158741716957
H	-1.14372967677478	-0.52975997296552	-0.02054061379730
H	-4.16042429608977	2.69351379822896	-1.60350558170313
H	-3.57856260478165	2.73922348604804	0.04702039702574
H	-1.56438907714567	-0.27019224562031	-2.84776805315993
H	-2.50625560684221	1.04672652224587	-3.56467450618856
H	-0.78810648333131	1.32022779110562	-3.10878650303404
H	-4.84768020062259	-4.14555972320789	-0.27433906380470
H	-6.03000969165506	-4.04449746059765	1.04137330731354
H	-4.29152713565446	-3.97759672018188	1.40959904905600
H	-6.08143610051392	-2.29259419730231	-1.39450585399011

H	-6.29662503723680	-0.69693278198015	-0.64508824710643
H	-7.19817126771782	-2.10366111908888	-0.03935556179967
H	-5.84526068710068	4.73848542530762	1.79511305715448
H	-4.42798820403511	3.65510706652927	1.80369418753867
H	-4.74615222943325	4.72291656697546	0.38723903811592

Table S6: Coordinates of epimer **4** in dichloromethane.

C	-5.31156543729326	-0.64515933359883	2.26744367352405
C	-4.81176065716228	-2.07846507871623	2.17299569719466
C	-4.12822958269861	-2.38503060467477	0.82866804068132
C	-2.99865211277612	-1.32636774672301	0.55750673575022
C	-3.24012196148841	0.18539525024390	0.91641187316915
C	-4.12310676492119	0.30061427412743	2.18422257847322
C	-2.42251380810003	-1.41669746979866	-0.85858336216917
C	-3.37222294419823	-0.86753413185564	-1.88352399849975
O	-4.18399236078311	0.11432279586388	-1.45017540392606
C	-3.80098001517151	0.97743706486610	-0.37874278926261
C	-1.87118692385188	0.75975712679423	1.34381846867859
C	-2.77395655799054	1.97326260499936	-0.94932062002395
O	-3.48802380618157	-1.26711153248809	-3.01214581901856
C	-5.05433584996661	1.80812831632820	-0.01581088074332
O	-4.89822387761319	2.81071966350434	0.64471720096591
C	-6.40432430165270	1.37182407038836	-0.50635500869288
C	-3.45767728528122	-3.76370289974516	0.94570806383041
C	-5.20824585402215	-2.49487001392139	-0.25902507490747
C	-3.30465123066642	2.80415015556990	-2.10146479757642
O	-4.45445695989156	2.83331945350865	-2.45943735555947
O	-2.41914834381216	3.56715522950177	-2.74889266046073
C	-1.04654650111308	3.63027916338350	-2.38399268080729
H	-5.82832866069015	-0.48649300798652	3.22754161775498
H	-6.05690127306794	-0.44028080666392	1.48365834615313
H	-5.63316532610667	-2.79828094770519	2.32556959839692
H	-4.08939695312868	-2.25214350199006	2.99122568948516
H	-2.19316523572607	-1.63167460866408	1.24387092128597
H	-3.46572242948613	0.04662853172083	3.03201046278587
H	-4.41537491781155	1.34576535991170	2.34649568363807
H	-1.48569723527441	-0.83963508862549	-0.92972862662722
H	-2.17159729594568	-2.44192199571743	-1.15884817426821
H	-1.95857658956676	1.80663518317272	1.67027310313320
H	-1.10274761481541	0.70932043880884	0.56070629105622
H	-1.49915125137040	0.18315592375342	2.20229984199487
H	-2.41514621833229	2.66124734925309	-0.17557121178913
H	-1.91113197840560	1.42149681300374	-1.35081391922463
H	-6.47185462129469	1.66351214504406	-1.56416525045568
H	-6.54486499550612	0.28578008624289	-0.45967992369894
H	-7.18223035699001	1.89073621513546	0.06791619519478
H	-2.98040439939636	-4.07688632833817	0.00402905852127
H	-4.20512232110918	-4.53174262341400	1.20129680308852
H	-2.68798347131813	-3.77040828670507	1.73431296660748
H	-5.94614219643821	-3.25549170722847	0.04088085616176
H	-4.79708715204242	-2.81923947465152	-1.22632456766879
H	-5.75277694015882	-1.55708201273530	-0.42384768492429
H	-0.59320261829637	4.38309934908916	-3.03975099450068

H	-0.54024793652542	2.66685110320021	-2.54670719713614
H	-0.91818928156050	3.94526568653123	-1.33802461358435

Table S7: Coordinates of epimer **3** in methanol.

C	-5.34803313005490	0.01241300784841	1.98370861636367
C	-5.46631503657445	-1.50232885913327	1.95957305720720
C	-5.12258791181924	-2.09702927767005	0.58359816158998
C	-3.69155735117897	-1.61879745157049	0.16236883021011
C	-3.24166709532981	-0.13488569956647	0.43399660862745
C	-3.90235617592800	0.41452387581775	1.72118599766983
C	-3.33246691520224	-1.99801146718503	-1.27353587008431
C	-4.00166577911310	-1.11168406592362	-2.27456824317707
O	-4.18164036730344	0.16643261695068	-1.91917109728773
C	-3.46764900113304	0.80515697549676	-0.85127806509331
C	-1.72980566445932	-0.21087006150244	0.75635488011285
C	-4.18982634782122	2.15417585318707	-0.62802101562221
O	-4.40842639471849	-1.46270324490358	-3.35787697401097
C	-2.11405186675679	1.30636815724771	-1.45997221962568
O	-1.46980460861715	2.13117386538774	-0.84983108989579
C	-1.69841172111106	0.83264007042892	-2.82119399015418
C	-5.08520209626045	-3.62648724483758	0.73120723141306
C	-6.23289528515302	-1.75327185550664	-0.41916813329415
C	-5.67482960607582	2.18804579064515	-0.35569064546751
O	-6.48414719108818	1.51474109207503	-0.95309341679753
O	-6.14232591643233	3.07329466763648	0.52364317651144
C	-5.32893670477778	4.02585294259593	1.20535373658232
H	-5.65059048486691	0.40520207002025	2.96772362757373
H	-6.04895659393083	0.44528035730896	1.25708066621935
H	-6.48298375413889	-1.82127385946898	2.24406866142880
H	-4.78120526925115	-1.92622932422417	2.71642534439526
H	-3.02774105779223	-2.21153137075194	0.80948027393618
H	-3.31389678755276	0.01343982086392	2.56261757950949
H	-3.76432979608729	1.50284133657910	1.78025020819980
H	-2.24512143734650	-1.90297011931533	-1.42849962367642
H	-3.58620979883057	-3.03472918279365	-1.52697009774369
H	-1.33529544298610	0.76092828672737	1.07936592450082
H	-1.57735792190480	-0.92554100804671	1.57696231072298
H	-1.12001771835764	-0.55265633183723	-0.09444789005394
H	-4.10095064115387	2.72734410788890	-1.56801572600165
H	-3.61375026779205	2.71314727556368	0.11321561828195
H	-1.56011103012792	-0.25817881346189	-2.84771698257010
H	-2.47422365111278	1.08215666233066	-3.56016222017167
H	-0.75490589965990	1.32247758205059	-3.09044665890720
H	-4.88295962827495	-4.13516738302374	-0.22436856797497
H	-6.05640441988580	-3.99808584812935	1.09611683272900
H	-4.31502096198337	-3.94400503750755	1.45277058744490
H	-6.09033697280768	-2.26991681039817	-1.38083264297845
H	-6.31627987353913	-0.67904026243121	-0.62365593611488
H	-7.20425248192972	-2.09133964658327	-0.02333236065981
H	-6.02108916330214	4.64481657995406	1.78822472020941
H	-4.62636520464496	3.53479007438823	1.89351944507482
H	-4.78654157383030	4.66589115677900	0.49513737084887

Table S8: Coordinates of epimer **4** in methanol.

C	-5.42741972256795	-0.04524058797134	1.98995187059728
C	-5.47435436561321	-1.56300354196715	1.95289587566906
C	-5.11761533230896	-2.12780990219592	0.56798923100598
C	-3.69928405425870	-1.60748310754837	0.15888770442321
C	-3.29961114878511	-0.11055713924245	0.43917310521815
C	-4.00850739543186	0.43514319330729	1.70628512224705
C	-3.31942363342951	-1.97641844896588	-1.27403829746467
C	-4.00011117195279	-1.10165615811509	-2.27732614219295
O	-4.18778687372553	0.17637516862896	-1.92827416448985
C	-3.47680667758339	0.82588804550307	-0.85226637254147
C	-1.79542616153892	-0.14409496294014	0.80795099189603
C	-4.21257539197953	2.17564281716719	-0.69079971465999
O	-4.40687733864574	-1.46218248040781	-3.35767902974349
C	-2.12180525761231	1.30809284626111	-1.47981230961743
O	-1.48906371648559	2.16876361165815	-0.90861857186906
C	-1.69087298838111	0.78492601991939	-2.81864549545352
C	-5.04621063184523	-3.65798696576517	0.69419681831353
C	-6.22963056931190	-1.79118574302226	-0.43550757866456
C	-5.65266880182973	2.20313168885075	-0.24480167757201
O	-6.51255285258407	1.51332147534314	-0.74623198474394
O	-6.02846891368541	3.07867497788768	0.68483473372327
C	-5.16803453309915	4.07823125403070	1.22993788728543
H	-5.72513378558618	0.32185889094538	2.98544924183750
H	-6.16401967565457	0.36028263921970	1.28443557935355
H	-6.47314656689459	-1.92940080785653	2.24362316609833
H	-4.76392041177879	-1.96521551250241	2.69835367868539
H	-3.02291435513068	-2.18097414503252	0.81052515083732
H	-3.39927219407613	0.10139348465259	2.56178694666435
H	-3.93639104346711	1.53130327540285	1.73512927394376
H	-2.23281940088070	-1.86023242311928	-1.41845047616344
H	-3.55052284371261	-3.01762039806340	-1.52996743341197
H	-1.43117708250321	0.84670583184420	1.11061382594094
H	-1.64893040733731	-0.83038229502715	1.65344952840686
H	-1.15607275758763	-0.49708739782812	-0.01644230252912
H	-4.23684194936646	2.63898714233392	-1.69214594756335
H	-3.57286071167701	2.81887670204161	-0.07961554212998
H	-1.54572741984239	-0.30459474483125	-2.80604046037004
H	-2.45737119897218	1.00663623585870	-3.57576039647042
H	-0.74638644724454	1.27003103511233	-3.09285496895940
H	-4.83256361432575	-4.15059441479101	-0.26716701219475
H	-6.00965374127814	-4.05456530656212	1.05338458116158
H	-4.27045078119778	-3.96835245372210	1.41300960932174
H	-6.08185166239965	-2.30852437055626	-1.39603289385068
H	-6.31557131501428	-0.71720807861676	-0.64110337208404
H	-7.20066088146815	-2.13239889613681	-0.04126037794470
H	-5.79207743131218	4.66506514990308	1.91391146365887
H	-4.33762702347584	3.63346846212620	1.79679118600580
H	-4.78242776516002	4.73837033478932	0.44037595038982

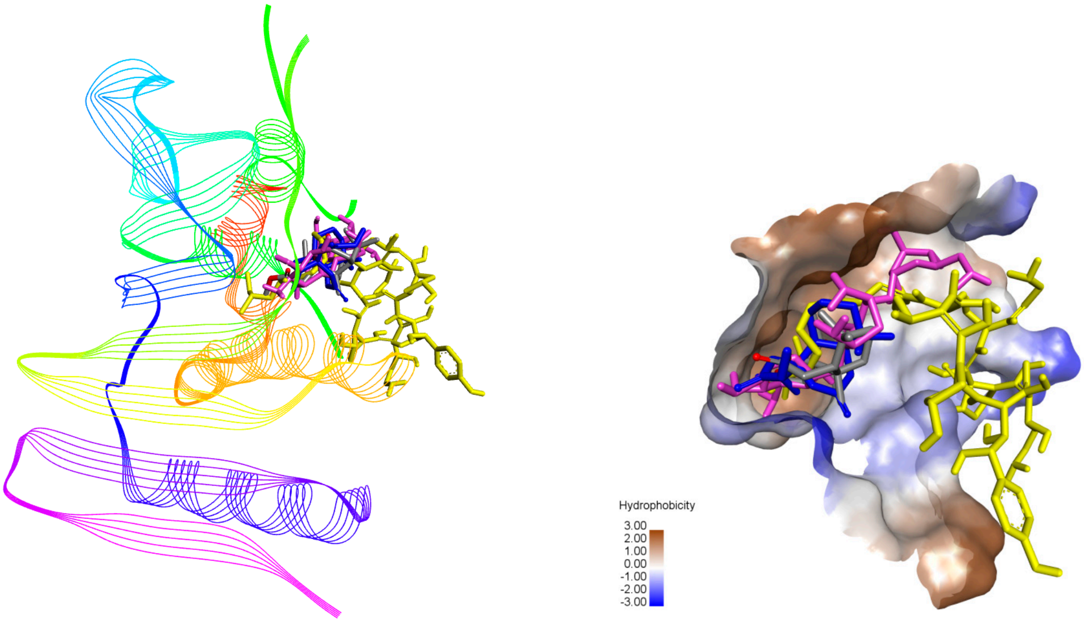

Figure S1: Positions of the best score conformations in the binding pocket of 1KZN (Caspofungin - yellow, Kanamycin - purple, compound **3** - blue, compound **4** - gray).

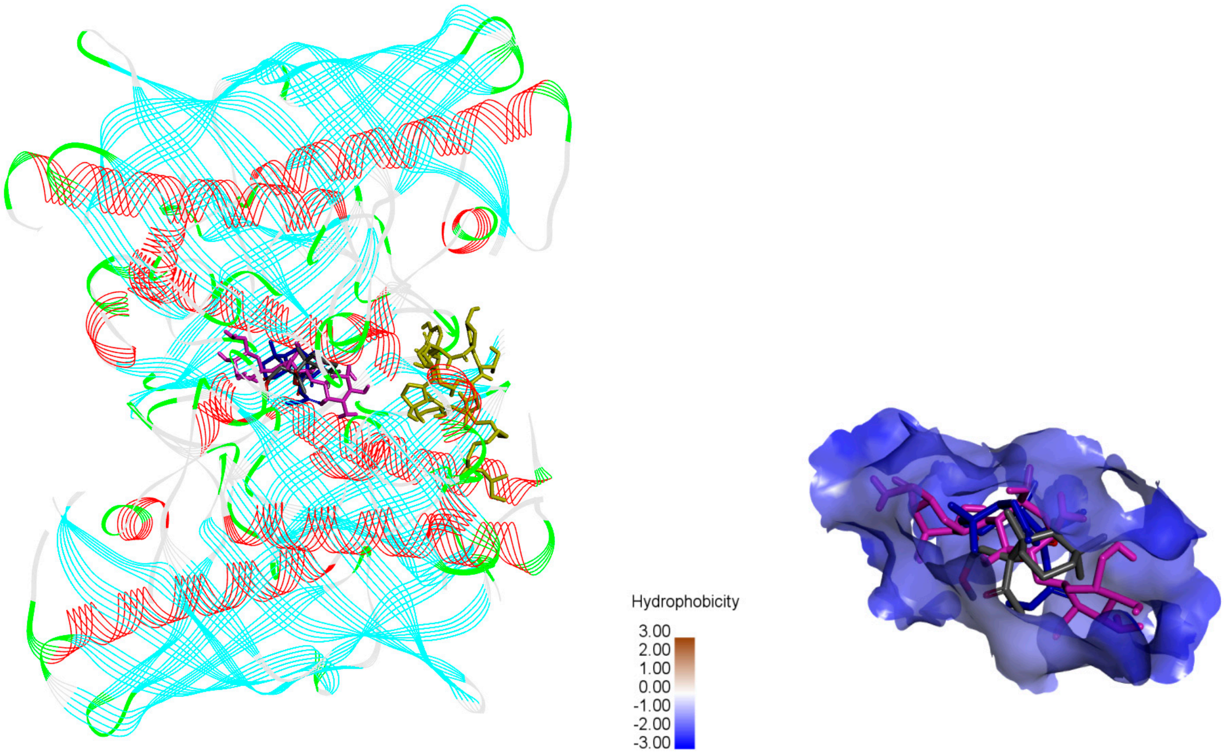

Figure S2: Positions of the best score conformations in the binding pocket of 1U1Z (Caspofungin - yellow, Kanamycin - purple, compound **3** - blue, compound **4** - gray).

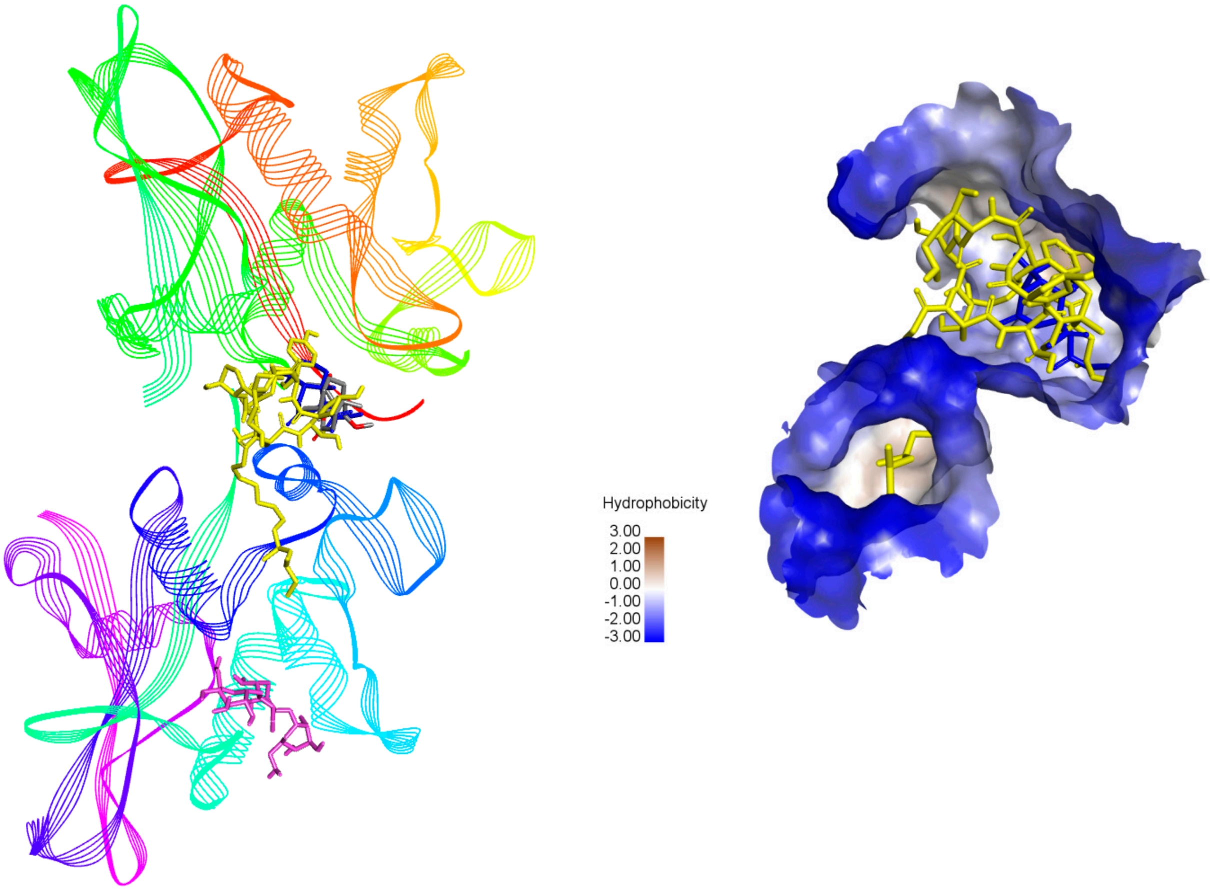

Figure S3: Positions of the best score conformations in the binding pocket of 3QLS (Caspofungin - yellow, Kanamycin - purple, compound **3** - blue, compound **4** - gray).

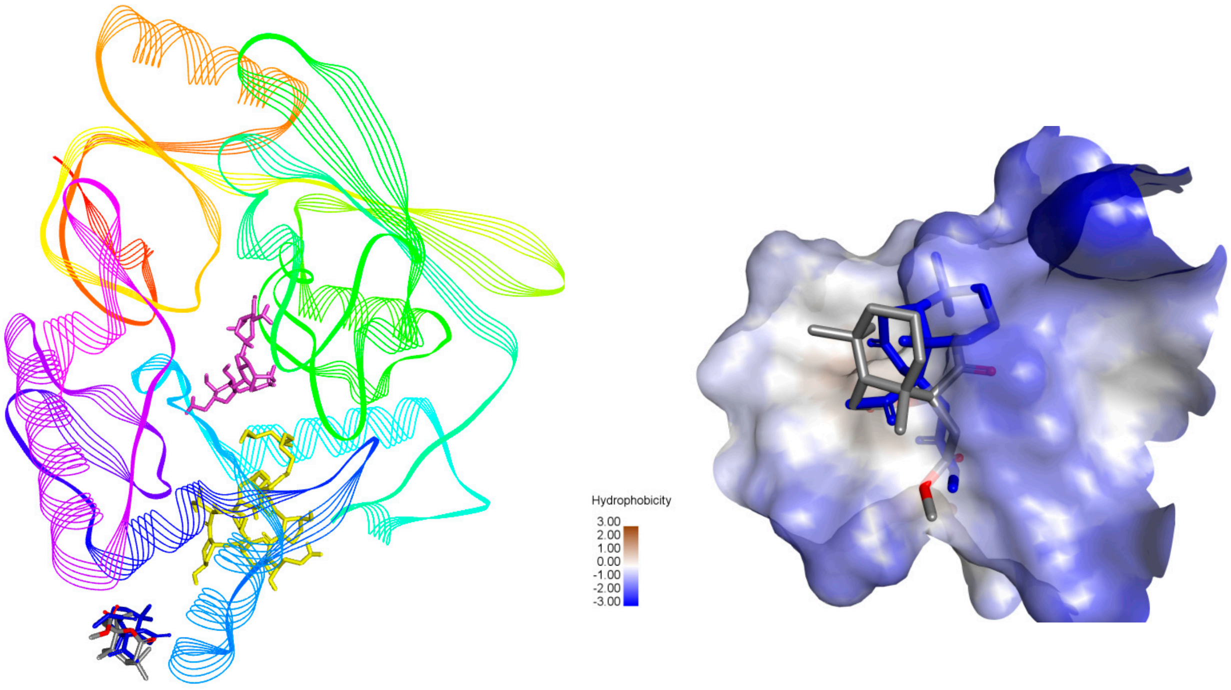

Figure S4: Positions of the best score conformations in the binding pocket of 2Q85 (Caspofungin - yellow, Kanamycin - purple, compound **3** - blue, compound **4** - gray).

Table S9: Crystal data and details of structure refinement for **4**, **5** and **6**.

Compound	**4**	**5**	**6**
Emp. formula	$C_{17}H_{16}O_5$	$C_{15}H_{24}O_3$	$C_{15}H_{24}O_3$
Fw	310.38	252.34	252.34
T [K]	293	298	200
space group	$P2_12_12_1$	$P2_1$	$P2_12_12_1$
a [Å]	8.8694(4)	7.7311(3)	7.5450(3)
b [Å]	10.4586(6)	11.1504(4)	7.7425(3)
c [Å]	17.5275(8)	8.5223(4)	24.2391(10)
α[°]	90	90	90
β [°]	90	110.499(5)	90
γ[°]	90	90	90
V [Å^3]	1625.89(14)	688.14(5)	1415.99(9)
Z	4	2	4
ρ_{calcd} [g cm^{-3}]	1.268	1.218	1.184
μ [mm^{-1}]	0.092	0.083	0.081
Crystal size [mm]	0.10 × 0.10 × 0.15	0.20 × 0.20 × 0.40	0.15 × 0.10 × 0.04
2Θ range	5.14 to 52	5.102 to 50.048	5.524 to 51.994
Refls. collected	6903	5298	11325
Indep. Refls., R_{int}	1838, 0.0633	2426, 0.0292	2790, 0.0592
Data/rests./params.	1838/0/204	2426/1/168	2790/0/168
GOF	1.018	1.072	1.025
R_1, wR_2(all data)	0.0488, 0.0865	0.0472, 0.1100	0.0554, 0.1096
CCDC no.	2253403	2255732	2257997

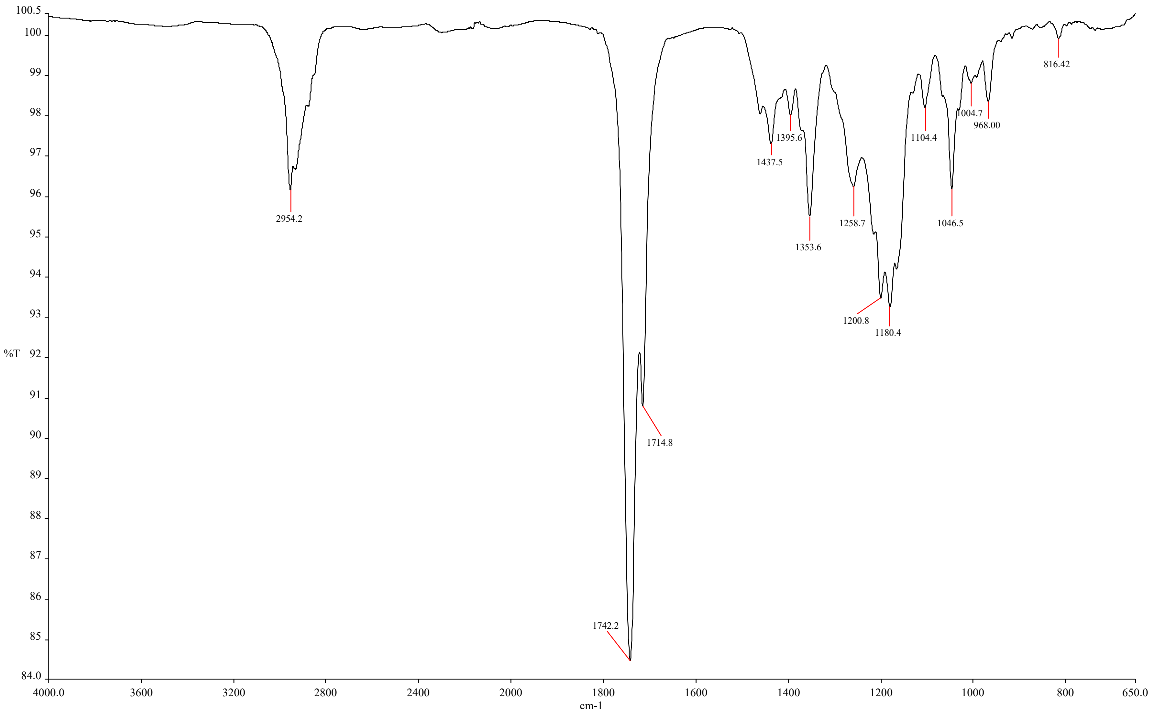

Figure S5: The IR spectrum of compounds **3** and **4.**

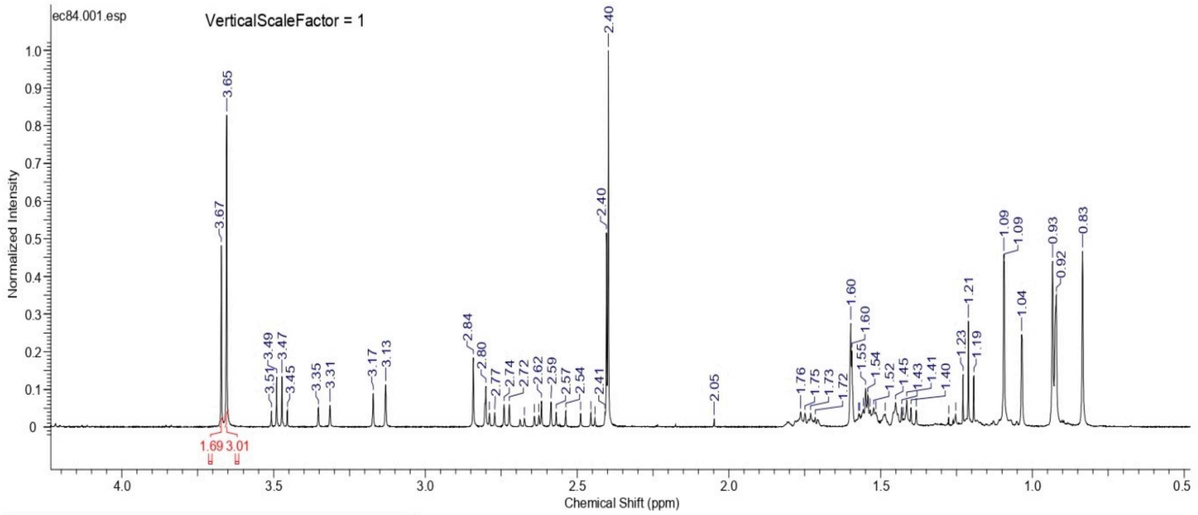

No.	(ppm)	Value	Absolute Value	Non-Negative Value
1	[3.6489 .. 3.65	3.00877953	3.20465894e+9	3.00877953
2	[3.6669 .. 3.67	1.68829429	1.79820659e+9	1.68829429

Figure S6: The ^{1}H NMR spectrum of compounds **3** and **4.**

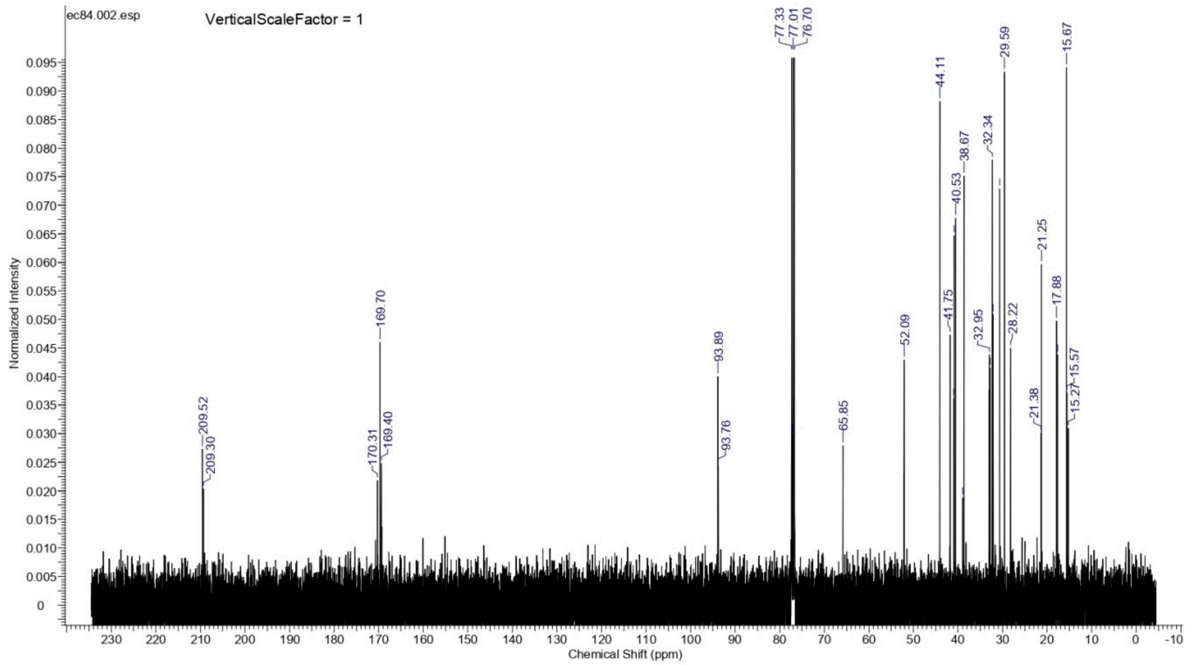

Figure S7: The ^{13}C NMR spectrum of compounds **3** and **4.**

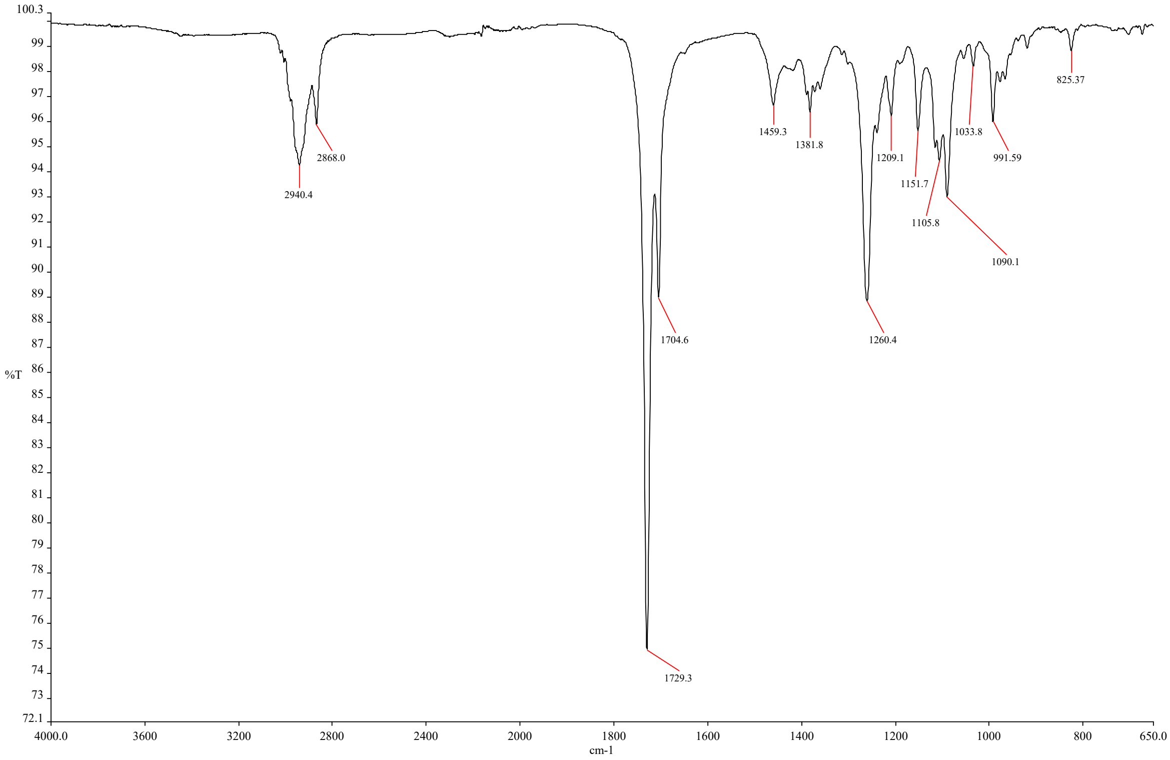

Figure S8: The IR spectrum of compound **5.**

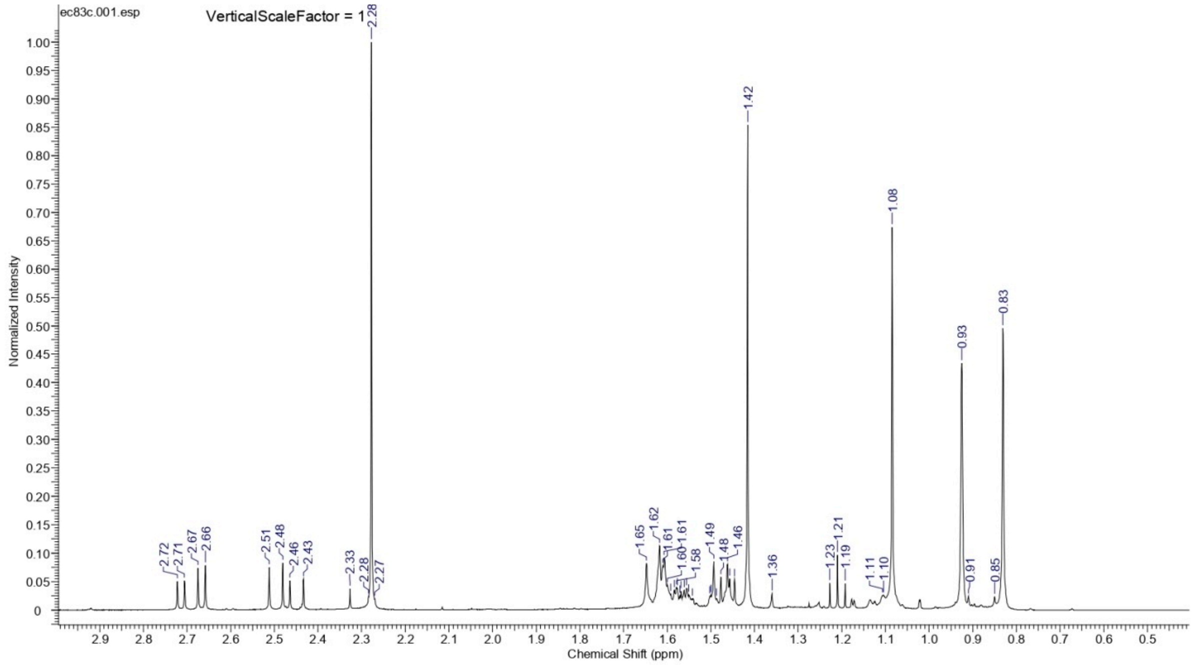

Figure S9: The ^{1}H NMR spectrum of compound **5.**

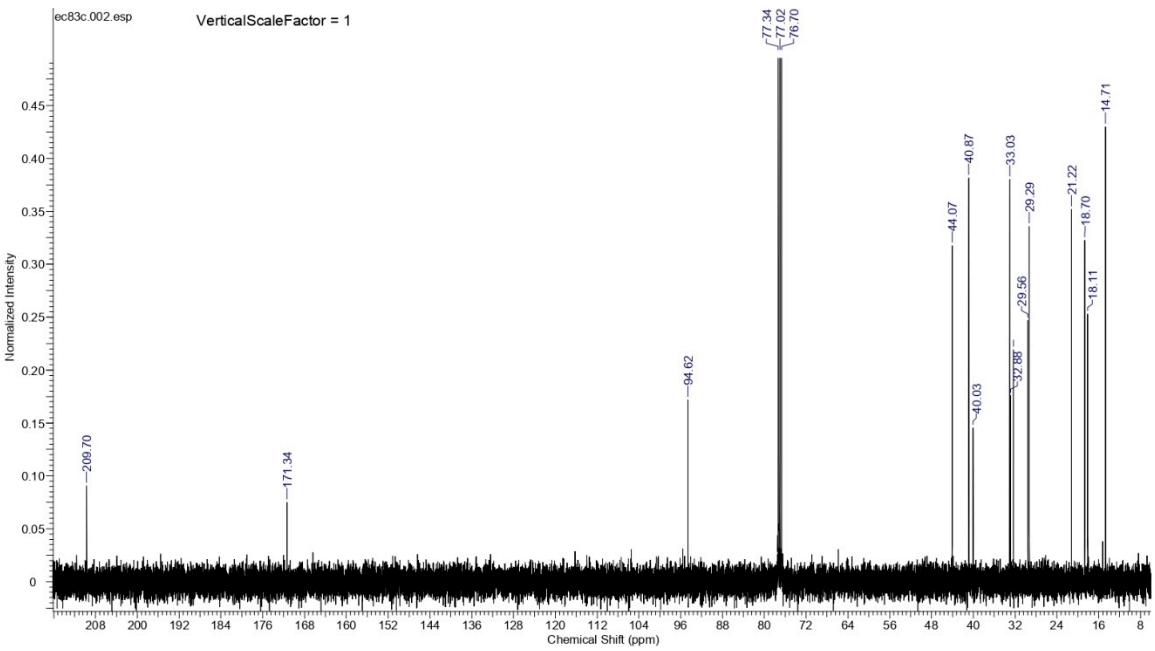

Figure S10: The ^{13}C NMR spectrum of compound **5.**

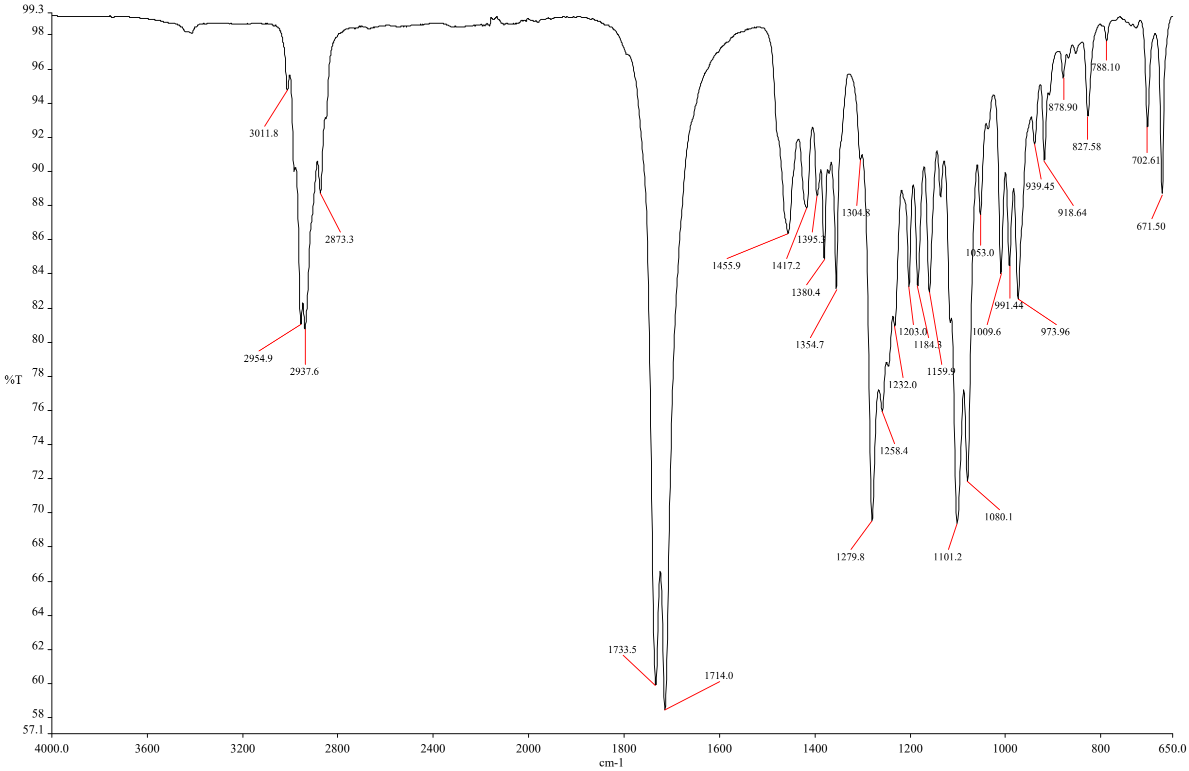

Figure S11: The IR spectrum of compound **6.**

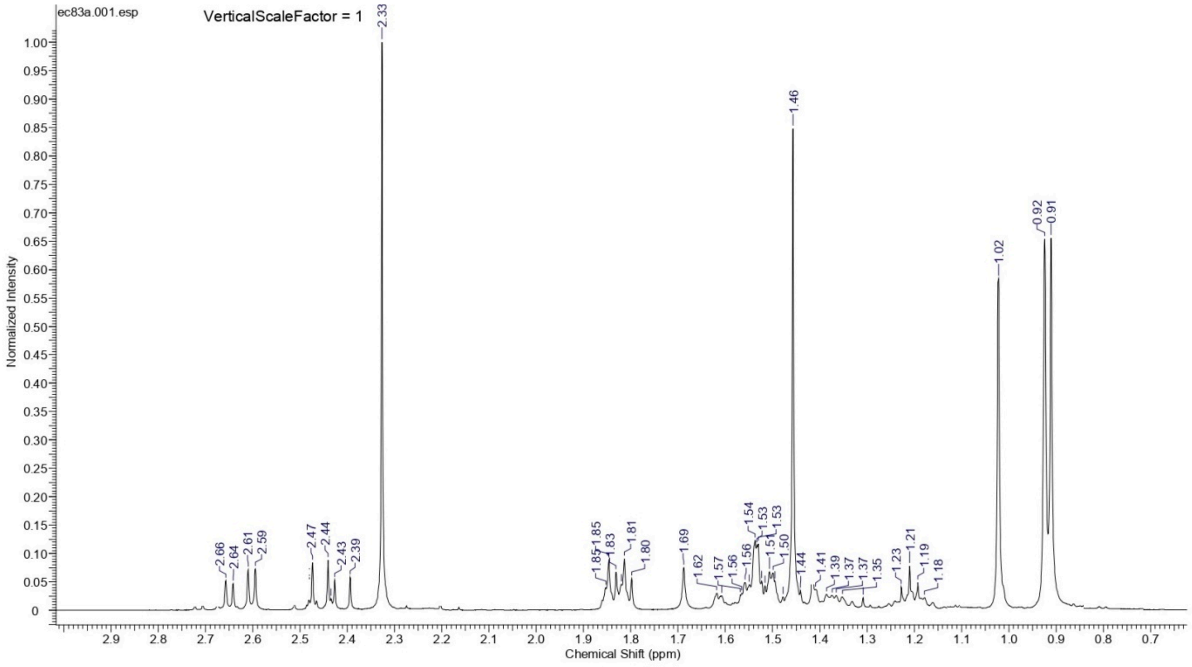

Figure S12: The ^{1}H NMR spectrum of compound **6.**

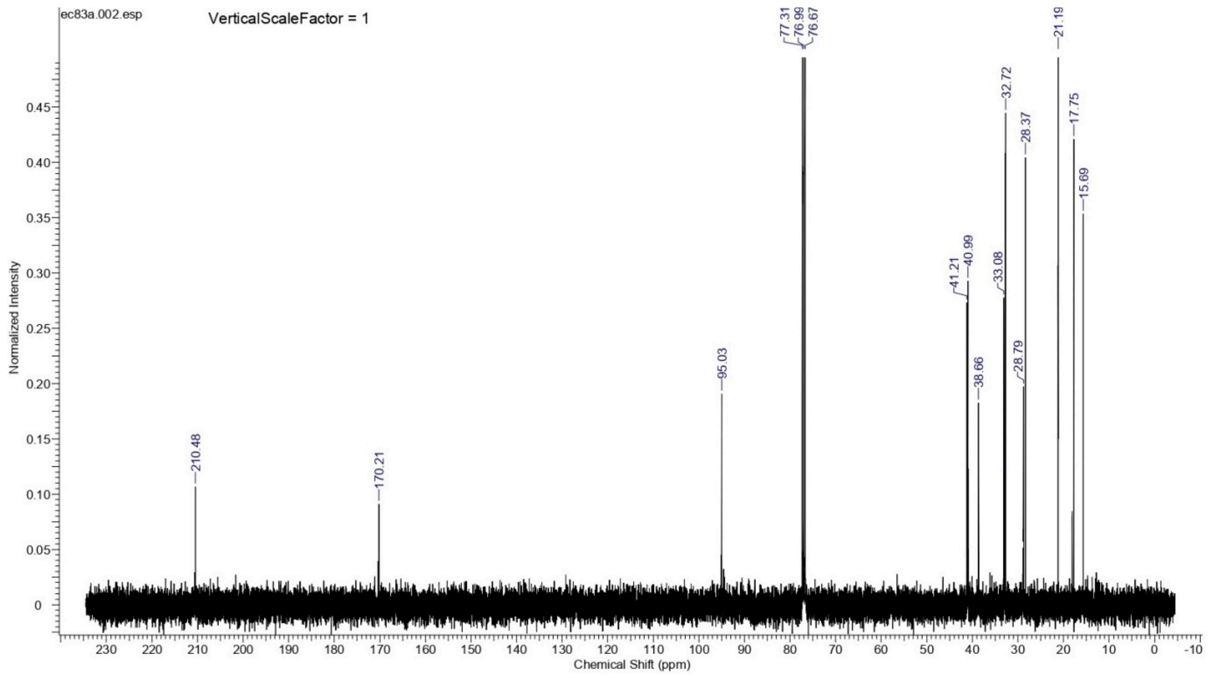

Figure S13: The ^{13}C NMR spectrum of compound **6.**
